# Understanding the Politics of Food Regulation and Public Health: An Analysis of Codex Standard-Setting Processes on Food Labelling

**DOI:** 10.34172/ijhpm.8310

**Published:** 2024-10-14

**Authors:** Monique Boatwright, Mark Lawrence, Angela Carriedo, Scott Slater, David McCoy, Tanita Northcott, Phillip Baker

**Affiliations:** ^1^Sydney School of Public Health, Faculty of Medicine and Health, University of Sydney, Sydney, NSW, Australia.; ^2^Institute for Physical Activity and Nutrition, School of Exercise and Nutrition Science, Deakin University, Geelong, VIC, Australia.; ^3^Department of Health, University of Bath, Bath, UK.; ^4^School of Exercise and Nutrition Science, Deakin University, Geelong, VIC, Australia.; ^5^International Institute for Global Health, United Nations University, Kuala Lumpur, Malaysia.

**Keywords:** Front-of-Pack Nutrition Labelling, Food Regulation, Codex, Conflicts of Interest, Public Health, Infants and Young Children

## Abstract

**Background:**

The importance of the international food regulatory system to global health, is often overlooked. There are calls to reform this system to promote healthy and sustainable food systems centred on the Codex Alimentarius Commission (Codex), the United Nation’s (UN’s) standard-setting body. Yet this presents a significant political challenge, given Codex has historically prioritized food safety risks over wider harms to public health, and is dominated by powerful food exporting nations and industry groups with a primary interest in trade expansion. To better understand this challenge, we examine who participates and contests Codex standards, using the development of the new Guidelines on Front-of-pack Nutrition Labelling (FOPNL) as a case study.

**Methods:**

The study involved: (*i*) collecting Codex Committee on Food Labelling (CCFL) documents (2016-2023); (*ii*) identification, categorization, and enumeration of actors involved in the development of the Guidelines; and (*iii*) guided by a constructivist framework, analysis of how actors framed and contested key provisions of the Guidelines.

**Results:**

Country representation was skewed towards high-income (47.9%). Member state delegations were dominated by non-health ministries (59.8%) and industry actors (16.1%). Industry actors comprised the large majority of observers (84.2%) and civil society actors representing public health interests the least (12.2%). Commercial actors used frames supporting positive FOPNL messages (eg, low in salt) opposing negative ones (eg, "high-in" sugar warnings) and called for product exemptions (eg, sports foods and baby foods). Public health actors used frames supporting simplified FOPNL to reduce consumer confusion, that hold up public health goals, and prevent inappropriate marketing.

**Conclusion:**

Participation in the Guidelines development process suggests stronger preferences for trade facilitation and commerce over public health. Ambitions to reform the international food regulatory system may require an examination of who participates and how to address this asymmetrical representation of interests. These results suggest the need to greatly strengthen public health representation at Codex.

## Background

**Key Messages**

**Implications for policy makers**

- To support food systems transformation, member states can examine and rectify the asymmetrical representation of high-income countries (HICs), non-health ministries, and industry groups in the development of Codex Alimentarius Commission (Codex) standards.
- This can involve ensuring public health interests, including ministries of health and food, nutrition, and health policy specialists, are given stronger representation within member state delegations; and that corporations and commercial interest groups are excluded or greatly reduced in the same delegations.
- Greater consideration can be given by member states to prioritize participation by civil society organizations representing public health and consumer interests in Codex standard-setting processes.
- Codex itself can ensure agenda items distributed prior to committee meetings include those pertaining to nutrition and public health. This will ensure fair attendance by civil society organizations and public health and nutrition policy specialists from member states, and opportunities to scrutinise industry proposals.
- Codex member states can take a more proactive approach to ensure the development of food standards and guidelines align with the current food systems transformation agenda, to give much more priority to public and ecological health.

**Implications for the public**

This research contributes greater understanding of the competing actors and interests involved in Codex Alimentarius Commission (Codex) food standard-setting processes. Our results highlight the need to strengthen participation by health ministries, lower-income countries and civil society organizations advocating for public health. Domination of the standard-setting process by industry interests, including trade-focused country delegates, raises the likelihood that without more thoughtful design, Codex deliberations may inadvertently exclude agenda items pertinent to nutrition and public health. This would preserve imbalances that preclude opportunities to rebut industry proposals that narrow the space for health-promoting regulation. This study also shows that pro-public health actors must be strategic to frame arguments promoting population health, empower countries to warn consumers about health risks through front-of-pack nutrition labelling (FOPNL) schemes, and avoid trade challenges.

Food systems are currently unhealthy and unsustainable with experts now calling for transformative change.^1-4^ This involves comprehensively redesigning a complex global food governance system, and includes one crucial yet often overlooked mechanism in public health and food systems scholarship—the international food regulatory system. This system incorporates the Codex Alimentarius Commission (Codex), as well as national and sub-national food regulatory bodies and standards.^5^

 Codex operates under the Joint Food and Agriculture Organization (FAO) of the United Nations (UN) and World Health Organization (WHO) Food Standards Programme, established 60 years ago.^5^ Today, risk assessment undertaken by food regulators in response to novel foods and additives is still largely concerned with food safety. Risk, as defined by Codex, is “a function of the probability of an adverse health effect and the severity of that effect, consequential to a hazard(s) in food”^5^ (p. 104)(eg, toxicological, microbiological, or food adulteration risk).Yet there is less emphasis on new public health and sustainability concerns (ie, harmful public health and ecological outcomes).^6,7^ In this paper, we recognize that the terms *risk* and *harm* have distinct meanings in food regulatory systems, yet actors often use these terms interchangeably during Codex standard-setting deliberations, and we therefore use them in the same way throughout this paper.

 One major global food systems’ challenge and risk or harm to public health and ecological outcomes, is the proliferation of unhealthy foods in human diets. Such “foods to limit or avoid” have been described in various ways in food-based dietary guidelines, predominantly in terms of risk nutrients, for example, as being high sugar, salt and fat foods.^8^ More recently, processing-related terms have been adopted, especially by countries across Latin America, including the term ultra-processed foods.^9^ This shift in terminology reflects a rapidly growing evidence base showing diets high in such foods are associated with multiple adverse health outcomes.^10,11^ Irrespective of terminology, the consumption of these foods is rising worldwide, including in many highly-populated low- and middle-income countries (LMICs).^1,3,12-15^ This dietary transition reflects the industrialization of food systems, urbanization, technological change, and globalization, including the marketing and political activities of transnational food corporations, and inadequate government policy responses to protect and promote healthy diets.^1,16^ Experts are now calling for urgent regulatory action to halt the rise of unhealthy foods in human diets, to drawdown consumption, and minimize harm.^2,17-19^

 Yet, with some exceptions,^20-23^ few studies have considered how food regulatory systems are responding to this issue, or whether the processes, technical guidelines and standards developed within existing food and nutrition regulatory systems, are enabling rather than curtailing, the proliferation of such foods. One important regulatory response is product labelling. The use of front-of-pack nutrition labelling (FOPNL) schemes as simplified interpretational aids, has substantially increased around the world, including stop-sign warning labels adopted throughout Latin America, Nutri-Score adopted across the European Union (EU), and the Multiple Traffic Lights label in the United Kingdom.^24^ And while FOPNL can help consumers make healthier choices, these schemes can also be exploited by manufacturers when nutrition and health claims attach health “halos” (eg, gluten-free) or favourable ratings to unhealthy products, as demonstrated by Australia’s Health Star Rating System.^24,25^

 Food labelling is an important component of a more comprehensive package of policy measures governments can implement to counter the spread of unhealthy foods, and yet the design and development of food labelling schemes is often strongly contested by industry and public health groups.^1,3,26^ To understand this better, in this paper we examine how different actors and interests contest issues raised during the amendment of the Codex Guidelines on Nutrition Labelling (CXG 2-1985)^27^ to include new Guidelines on FOPNL.

 The development of the CXG 2-1985 and FOPNL Guidelines is overseen by the Codex Committee on Food Labelling (CCFL),^28^ which is responsible for drafting labelling provisions applicable to all foods and revising, amending, and endorsing those prepared by the other Codex Committees. Altogether, the CCFL oversees five guidelines, a compilation of Codex texts relevant to the labelling of foods derived from modern biotechnology, and three standards—the General Standard for the Labelling of Prepackaged Foods, the General Standard for the Labelling of and Claims for Prepackaged Foods for Special Dietary Uses, and the General Standard for the Labelling of Non-retail Containers of Foods.^28^ The CCFL is also linked to other committees, such as the Codex Committee on Nutrition and Foods for Special Dietary Uses,^29^ which includes standards for processed food products for infants and young children and foods for special dietary uses (FSDU), including sports foods and beverages (hereinafter referred to as sports foods).

 One of the terms of reference for the CCFL is “to study problems associated with the advertisement of food with particular reference to claims and misleading descriptions.”^30^ The delivery of information through food labelling is powerfully shaped by the political nature of food regulation.^31^ A growing body of scholarship shows that such regulations are often contested by different actors and coalitions, including industry, governments, and civil society organizations, often with competing interests concerning trade promotion versus public health and consumer protection.^2,3,20-23,32-39^ The transnational food industry itself is dominated by manufacturers headquartered mainly in and with close ties to the governments of the United States and EU countries, as well as a global network of trade associations who promote international and national food regulations conducive to market growth and profitability.^2,3^ Yet, with some exceptions,^20,22,23,32-34^ limited studies have investigated the contestation of Codex standards, including who is involved, how, and in response to which issues and whom.

 Using the development of new FOPNL Guidelines as a case study, our aim in this paper is to understand the politics of food standard-setting in Codex, and the challenges associated with regulating in the interests of public health. We adopted the following two objectives: (*i*) to quantify the government, industry and civil society actors involved in the development of the new FOPNL Guidelines; (*ii*) to understand how different actors and interests framed and contested these Guidelines, in response to which issues and whom, and to understand their interpretation of risk or harm during deliberations.

## Methods

 Given the complex nature of the topic, we adopted a case-study design incorporating quantitative and qualitative components.^40^ First, we enumerated the actors and interests involved in the development of the FOPNL Guidelines from 2016-2023, by quantifying country delegates and observers who participated in relevant CCFL meetings. Second, we conducted a theoretically guided framing analysis to understand how actors contested key provisions of the FOPNL Guidelines.^41-43^ To further contextualise the case study, we provide more background in the section below.

###  Context and Setting of the Case Study

 Codex was established in 1963 and operates with the dual mandate of protecting consumer health and facilitating fair practices in food trade.^5^ Today, Codex has 189 members, including the EU,^44^ and 235 observer organizations who participate in the development of international food standards that comprise the Codex Alimentarius.^45^ Member states typically include representatives from government, industry, civil society, and academia in their delegations, with national policy determining which actor types can participate.^5^ When voting for or against a proposal, each member state delegation is permitted a single vote.

 Observers can also participate upon the advice of the Executive Committee. They include UN member states without Codex status, intergovernmental organizations including the WHO, and international non-governmental organizations (NGOs). Although observers have no voting rights, they can influence decision-making by engaging in dialogue with other delegates before and between sessions, circulating research or conference room documents, and may be asked to address a committee. Participation by delegates and observers contributes to the development of standards, guidelines and codes of practice adopted by Codex.^5^ Although voluntary, Codex documents inform national food regulations and legislation, particularly in LMICs where they are often adopted verbatim.^20^

 Since 1995, Codex as the standard-setting body, and Codex standards, have been recognized in legally binding trade and investment agreements of the World Trade Organization (WTO). Because of this, when countries implement national standards exceeding those set by Codex, they may be subject to challenges under international trade law.^20,23,46-50^ Given this growing importance to international trade law, Codex has become increasingly politicized, with government ministries, industry groups, and civil society organizations becoming increasingly active in Codex standard-setting processes.^20-23^ An example is the development of Codex FOPNL Guidelines.^27^

 According to Codex, FOPNL is, “…a form of supplementary nutrition information that presents simplified, nutrition information on the front-of-pack of pre-packaged foods…”^27^ (p. 11).Discussions on FOPNL began at Codex following the 2015 WHO* Technical Meeting on Nutrition Labelling for Promoting Healthy Diets*. In 2016, at the CCFL43 session, Costa Rica, the Dominican Republic, and Uruguay submitted a discussion paper on harmonization principles and FOPNL,^51^ followed by a proposal by the International Association of Consumer Food Organizations (IACFO) for new work on a global standard for interpretive FOPNL,^52^ and a proposal by Costa Rica and New Zealand (NZ) for Codex to provide guidance on FOPNL.^53^ Codex agreed to examine the different FOPNL systems and develop high level principles to inform a new standard or expand on CXG 2-1985. An electronic working group chaired by Costa Rica and NZ was then appointed to develop new FOPNL Guidelines aimed at maximizing global harmonization of voluntary and mandatory labelling schemes, to reduce trade barriers, provide consumers with simplified nutrition information, encourage the reformulation of food products, and support participation by Codex members.^53^

###  Categorization of Codex Participants

 To address the first objective, we identified, categorized and enumerated actors participating in annual CCFL meetings (2016-2023) as member state delegates and as observers, using data from annual meeting reports. For each actor category we reported the mean number and proportion of the total number of participants in each meeting.^54-59^ Member state delegations were categorized as either a high-income country (HIC), upper-middle-income country (UMIC), LMIC, or low-income country (LIC), as determined by gross national income per capita.^60^

 We separated delegations into sub-groups—health ministry, non-health ministry (agriculture, commerce, and trade etc), civil society organizations (including consumer and public health groups), food industry (food organizations and consumer groups representing food manufacturers), and others (EU Commission, embassy personnel, Codex secretariat, and academics etc). Additionally, there are 200 approved observers who attend meetings and circulate documents.^5,61^ Observers are formally categorized as non-member countries, intergovernmental organizations (entities formed between and receiving financial and political support from two or more nations), or NGOs (entities typically independent of government).^62^ We divided NGO observers into two groups—civil society organizations (independent of government and industry affiliations) and industry (independent of government, privately funded, typically with food industry affiliations).^62,63^

 Our results excluded data from 2018, 2020, and 2022 because CCFL meetings were not held due to the COVID-19 pandemic. In 2021, meetings were held virtually.

###  Qualitative Framing Analysis

 To understand how actors framed and contested relevant issues, we adopted a constructivist approach and theoretically-guided framing analysis method.^42,64^ This involved three steps: (*i*) document collection, entailing a search for Codex documents relating to the development of the FOPNL Guidelines; (*ii*) developing a theoretical framework to guide the analysis; and (*iii*) coding of the textual data and refinement to generate a final set of themes.

####  Document Screening and Selection

 The initial document search applied key words and phrases to identify which Codex committees discussed issues relevant to the regulation of unhealthy packaged foods. Terms used included unhealthy, nutrition, process*, ultra-process*, claims, and label*. Documents were scanned by the lead author to determine which issues generated substantial contestation and which years were most suitable to analyze in greater depth. We decided to focus the case study on the development of the new FOPNL Guidelines led by the CCFL, given the availability of documents, the participation of diverse actors, and obvious contestations over several issues. The search terms were then reapplied along with the terms front-of-pack and labelling to CCFL documents (2016-2023) on the Codex website.^65^ The inclusion and exclusion criteria was applied, as shown in Table S1 (Supplementary file 1), and the final set of documents for analysis was approved by group discussion.

####  Framing Analysis

 All relevant Codex documents (n = 60) were uploaded to the qualitative analysis software NVivo (QSR International, version 12.5) and were coded, using a coding schema derived from the framework outlined below (Table 1).^66^ This framework was adapted from earlier studies on the framing of obesity prevention and food regulation.^22,67-69^ This framework was developed using a constructivist approach where the “frame” is a unit of analysis, defined as a central organizing principle that “governs the subjective meaning [assigned] to social events.”^41-43^ Frames can be deployed by actors to portray problems in ways that attract or conceal attention, portray risk or harm, counter opposing frames, and further their interests.^41,43,70-72^ We designated actor preferences as aligning with commercial or public health interests based on their framing of causation, responsibility, and solutions. As the development and revision of Codex documents involves a highly structured and technical process, actors often drew on evidence to support their perceptions of risk, harm, and solutions throughout the Guideline development process.^73-75^

**Table 1:** Framework Used to Guide the Framing Analysis

**Categories**	**Sub-categories**	**Prompts for Coding**
Interests	Commercial interests Public health interests	What are the inherent interests represented by the responses to proposals?
Frames	Causation	What/who is identified as the cause of the problem? How is “risk/harm” perceived?
	Responsibility	Who is responsible for addressing the problem?
	Solutions	What are the policy actions to mitigate the problem? What are the potential benefits and pitfalls of those actions?
	Evidence	What evidence (if any) is used to support positions?
Outcome		What was the outcome of the process involved in developing the new Codex FOPNL Guidelines?

Abbreviation: FOPNL, front-of-pack nutrition labelling.

## Results

 The results are structured in two sections. The first, categorizes and enumerates actors participating in CCFL meetings. The second, presents how actors contested and framed issues during the development of the FOPNL Guidelines.

###  Quantitative Component – Enumeration of Actors 

**Figure 1:**
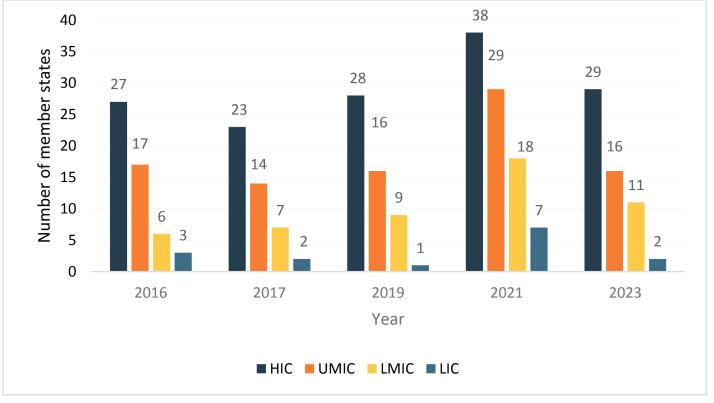
Number of Member State Delegations Participating in CCFL Meetings Categorised by Country Income Category From 2016 to 2023. Abbreviations: HIC, high-income country; UMIC, upper-middle-income country; LMIC, low- and middle-income country; LIC, low-income country; CCFL, Codex Committee on Food Labelling. Note: Countries were classified by World Bank category, based on gross national income per capita data (Atlas method) for the previous calendar year.^60^ The CCFL did not meet in 2018, 2020, or 2022.

Figure 1 quantifies member state delegations participating in CCFL meetings from 2016 to 2023, grouped by country income category. During this period, 61 countries on average were at each meeting, with participation by HICs the greatest. Overall, the average number of representatives per delegation decreased in relation to country income category—HICs had the highest mean number and proportion of the total number of delegates (mean = 29, 47.9%), UMICs (mean = 18.4, 30.4%), LMICs (mean = 10.2, 16.8%), and LICs had the lowest (mean = 3, 4.9%). To put this in context, of the 217 countries in the world in 2016, 31 (14.3%) were LICs, which was substantially more than 3 (5.6%) LICs of the 53 Codex member states represented in CCFL meetings.^76^

**Figure 2:**
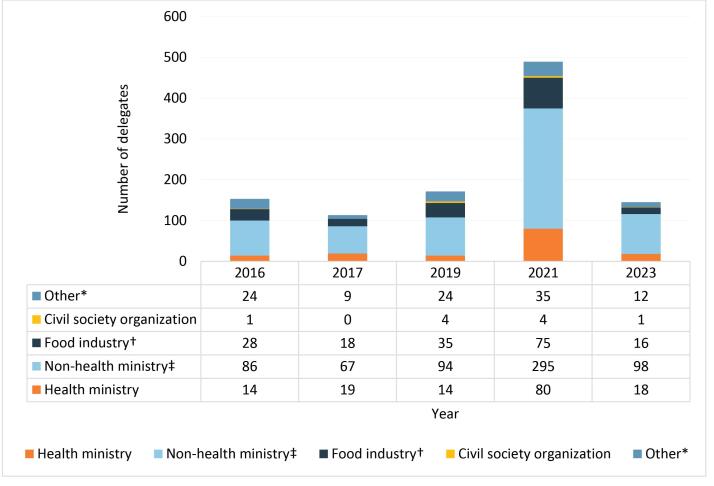
Number of Delegates by Actor Type Participating in Member State Delegations at CCFL Meetings From 2016 to 2023. Abbreviation: CCFL, Codex Committee on Food Labelling. Notes: *Other includes embassy personnel, national Codex representatives, academics, hospital personnel, lawyers, and undisclosed delegates. † Food industry includes all food manufacturers, and associations representing manufacturers and industry-sponsored consumer organizations. ‡ Non-health ministry includes agriculture, commerce, trade, and food and drug standards bodies. The CCFL did not meet in 2018, 2020, and 2022.

Figure 2 shows the actor types represented in member state delegations each year. Government officials were in the majority, with higher non-health ministry (mean = 128, 59.8%) compared to health ministry representation (mean = 29, 13.5%). Non-health ministries included agriculture, commerce, trade, and food and drug standards bodies. Of the non-government actors represented in delegations, industry representation was higher (mean = 34.4, 16.1%) compared to “other” (mean = 20.2, 9.4%) and civil society organizations (mean = 2, 0.9%). In 2021, when committee meetings were held virtually, participation increased by ~50% for most actor categories, other than civil society organizations. Industry representation within delegations increased each year until 2023 when it dropped by ~150% from the previous year. Between 2016 and 2023, six countries on average selected industry actors to represent half or more of their delegation. Costa Rica, Mozambique, and Malaysia had industry as their sole representative.

**Figure 3:**
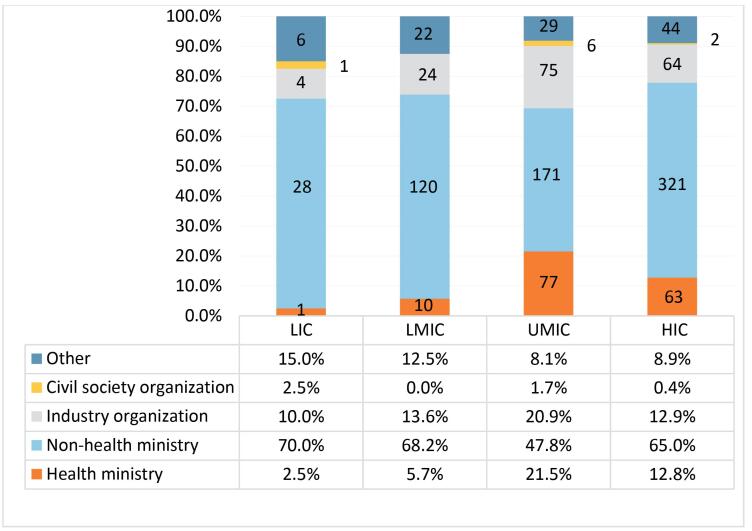
Proportion of Actor Types Represented in Member State Delegations at CCFL Meetings by Country Income Category From 2016 to 2023. Abbreviations: LIC, low-income country; LMIC, low- and middle-income country; UMIC, upper-middle-income country; HIC, high-income country; CCFL, Codex Committee on Food Labelling.

Figure 3 shows the proportion of actor types represented in member state delegations organized by country income category between 2016 and 2023. Irrespective of country income level, delegations were dominated by representatives of non-health ministries. Delegations of HICs and UMICs had higher representation of health ministries, compared with LICs and LMICs. Industry groups were strongly represented in delegations from all country income levels, but especially in those of UMICs and HICs. In contrast, civil society organizations were barely represented, and were completely absent from LMIC delegations.

**Figure 4:**
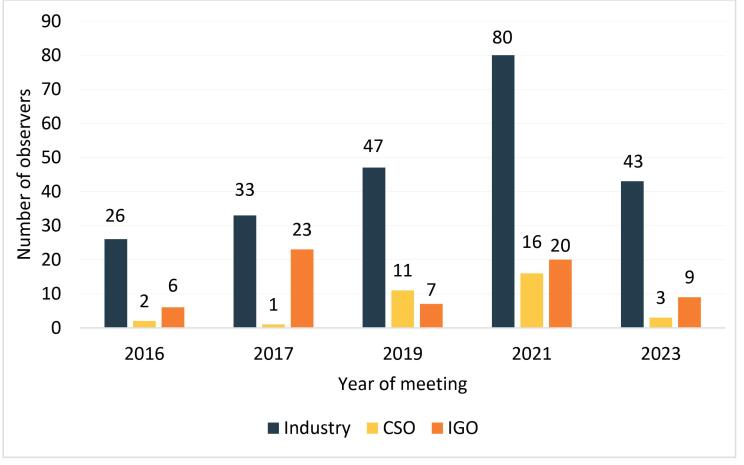
Number of Observers by Actor Category Participating in CCFL Meetings Between 2016 and 2023. Abbreviations: CSO, civil society organization; IGO, intergovernmental organization; CCFL, Codex Committee on Food Labelling.

Figure 4 shows the number of observers by actor type who participated in CCFL meetings between 2016 and 2023. On average, during this period, industry actor representation was significantly higher (63.1%) than intergovernmental organizations (27.8%) and civil society organizations (9.1%). In all years, except 2017, industry representation was at least four-times greater than representation by the other respective categories.

 Table S2 (Supplementary file 2) shows the main industry actors represented in member state delegations at CCFL meetings, including those of 14 HICs, 19 middle-income countries and 4 LICs. These actors include transnational food companies involved in the supply of food ingredients and packaged food manufacturing, as well as international and national trade associations, and industry affiliated science communication organizations. Between one and 15 of these industry actors were represented in member state delegations, with Thailand permitting the most (n = 15), followed by Mexico (n = 8) and the United States (n = 6). Certain transnational food corporations, headquartered in the United States and Europe, were represented in delegations across all country income categories, including Abbott (5 HICs, 12 UMICs/LMICs), Coca-Cola (6 HICs, 10 UMICs/LMICs), Reckitt Benckiser (3 HIC, 2 UMICs/LMICs), and Nestlé (5 HICs, 4 UMICs/LMICs, 1 LIC).

 Table S3 (Supplementary file 3) shows the frequency and names of NGOs participating as observers at CCFL meetings from 2016 to 2023. Industry organizations were nearly three-fold more frequent (n = 34) than civil society organizations (n = 12). Seven industry organizations participated in all five years analyzed, including the prominent international trade associations—the International Council of Beverages Associations, the International Council of Grocery Manufacturers Associations, and the International Dairy Federation (IDF/FIL). No civil society organization participated in all five years, although three participated in three years—the International Baby Food Action Network, the IACFO, and the World Obesity Federation.

###  Qualitative Component – Framing Analysis

 This section presents the results of the framing analysis, organized by how actors framed risk or harm when responding to proposals and contesting provisions for new Codex work on FOPNL Guidelines during and between annual CCFL meetings from 2016 to 2023. The interests of actors, and how they framed their arguments, is presented in Table 2. Although generally all actors supported the development of new FOPNL Guidelines, there were divergent commercial and public health views on the labelling schemes, including how nutrients were depicted on labels, and the implications of FOPNL requirements for certain product categories, such as sports foods and FSDU.

**Table 2:** How Actors Framed Arguments During Contestations of the FOPNL Guideline Provisions During CCFL Meetings From 2016 to 2023

**Interests**	**Actors**	**How Arguments Were Framed**
**Provision 1: FOPNL Schemes and Depiction of Nutrients**		
Commercial interests	Costa Rica, Dominican Republic, Uruguay, NZ, EU, Paraguay, Russian Federation, Australia (eWG reported 13 countries), IDF/FIL, FoodDrinkEurope, IFT, IFU, ICGMA, ISDI.	*Causation: what or who is the cause of the problem and how is risk perceived?*
		• There are adverse economic, trade and social implications of diverse FOPNL schemes; harmonization is desirable. • Labels depicting unhealthy nutrients with negative connotations (eg, “high-in” warnings) can be harmful to trade and do not provide constructive nutritional help. • Non-scientifically grounded FOPNL schemes can cause unjustified discrimination against products. • Misleading/alarmist graphics and verbiage can cause unfair discrimination against certain products. • Directive/specific guidelines limit the potential innovation of FOPNL schemes. • FOPNL schemes can discriminate against FSDU.
		*Responsibility: who is responsible for addressing the problem?*
		• Codex guidelines. • CCFL eWG. • Codex member governments.
		*Solutions: what are the policy actions to mitigate the problem?*
		• Developing harmonization principles via Codex FOPNL Guidelines. • Aligning with existing regulations (eg, EU regulations). • Adopting provisions that raise dietary awareness through positive nutrient profiling (eg, “low-in” salt). • Adopting provisions with meaningful nutritional information that reflect the dietary needs of consumers (eg, FSDU). • Adopting provisions that consider the overall nutritional value of a food product based on scientific evidence. • Keeping FOPNL Guidelines broad to cover various food products to suit different consumer needs. • Maintaining voluntary industry requirements.
		*What are the potential harms of those actions?*
		• Proliferation of additional inconsistent regulations that hinder trade. • Consumers making healthier purchasing choices.
		*Evidence: What evidence is used to support this position?*
		• Scientific principles used in CXG 2-1985. • WTO challenges.
*Summary of commercial interest themes of perceived risk or harm:* Economic implications, trade implications, fear of exploitation, discrimination, consumer misconceptions, regulatory restrictions.		
Public health interests	Canada, Chile, Ecuador, El Salvador, Guatemala, Honduras, Peru, South Africa, Uruguay, BEUC, CI, ENCA, HKI, IACFO, IBFAN, UNICEF, WFPHA, WOF.	*Causation: what or who is the cause of the problem and how is risk perceived?*
		• Codex standards do not include provision for simplified nutrition labelling that can be easily understood by consumers, support healthier choices, and reduce NCD risk. • By using general terms to describe agenda items, nutrition policy specialists may refrain from drawing upon their resources to attend specific CCFL meetings. • By not including comments made in Codex conference room documents in annual CCFL reports, issues relating to nutrition and public health concerns may not be adequately addressed in subsequent meetings. • FOPNL schemes used in LICs can create trade barriers with HICs that use different labelling schemes. • To ensure consistency and policy coherence, Codex documents should consider interpretive and simplified labelling to be FOPNL. • Design details on FOPNL, such as “positive” nutrient declarations, could encourage the use of “health halos,” whereby an unhealthy product may be seen by consumers as being healthy. • If the scope of the FOPNL Guidelines is too narrow, this could lead to the inappropriate promotion of “risky” products, such as breastmilk substitutes. • Unless FOPNL warnings are made mandatory, the food industry is unlikely to comply with labelling that highlights negative properties of manufactured products.
		*Responsibility: who is responsible for addressing the problem?*
		• Codex guidelines. • Codex systems and procedures. • Codex member governments. • WTO agreements.
		*Solutions: what are the policy actions to mitigate the problem?*
		• Improving Codex standards and FOPNL Guidelines. • Prioritizing nutrition and human health items on meeting agendas and including descriptive titles to ensure attendance by nutrition policy specialists. • Improving member and observer attendance at CCFL meetings to advocate for nutrition and public health. • Facilitating harmonized FOPNL schemes through Codex guidelines to remove inconsistencies and barriers to trade. • Including warning labels in the Codex definition of simplified FOPNL to inform consumers “at-a-glance.” • Consistently referring to WHO guidance during the development of Codex documents. • For the scope of FOPNL to be determined by the product type (eg, the requirement of warning labels for foods for infants and young children). • Mandatory FOPNL as an industry requirement for unhealthy packaged foods.
		*What are the potential harms of those actions?*
		• Resources may not be available for optimal systemic processes or meeting attendance. • Overarching FOPNL guidelines could permit “positive” nutrients to be exploited. • Codex guidelines could reflect commercial priorities that undermine public health.
		*Evidence: what evidence is used to support this position?*
		• CCFL43 agenda.^77^ • WHO reports, guidelines and technical meetings.^52,78,79^ • WTO meetings.^52^ • FAO and PAHO (ie, support of Chile’s “stop sign” warning label).
*Summary of public health interest themes of perceived risk or harm:* Commercial determinants of health, power asymmetries, systemic barriers, exclusion from decision-making, inconsistent labelling schemes, barriers to NCD prevention, voluntary regulations.		
**Provision 2: FOPNL – Requirements for Certain Food Products**		
Commercial interests	Argentina, Australia, Costa Rica, EU, NZ, US, ESSNA, Food Industry Asia, IFT, ISDI.	*Causation: what or who is the cause of the problem and how is risk perceived?*
		• There are Codex standards already in place for FSDU, which means these products should be exempt from FOPNL requirements. • Exempting some products from FOPNL requirements (eg, gluten-free foods) is deceptive and prevents consumers from exercising free choice. • Whether or not sports foods should require FOPNL should be government led to accommodate the different definitions of sports foods in different countries.
		*Responsibility: who is responsible for addressing the problem?*
		• Codex standards and guidelines. • Member states and voluntary industry regulations. • Individual consumers.
		*Solutions: what are the policy actions to mitigate the problem?*
		• Excluding FSDU from FOPNL requirements. • Including gluten-free foods in FOPNL requirements. • Domestic regulations. • Voluntary industry regulations. • Following Codex food standards.
		*What are the potential harms of those actions?*
		• FOPNL could be used to promote FSDU; this opportunity could be lost if products are exempt from FOPNL. • Some countries may implement mandatory and/or robust FOPNL (eg, ‘stop sign’ warning labels). • Voluntary FOPNL may encourage consumers to make healthier choices. • If relevant Codex standards do not exist, food industry may be required to provide consumers with dietary guidance to help them make an informed decision.
		*Evidence: what evidence is used to support this position?*
		•Codex standards and guidelines.
		• WHO guidelines.^79^ • Voluntary FOPNL systems (eg, Australia’s HSR). • National legislation (eg, EU legislation and General Food Law).
*Summary of commercial interest themes of perceived risk or harm: *Directive and specific regulations, prevents consumers from exercising free choice, and government led schemes.		
Public health interests	African Union, Canada, Kuwait, Nigeria, South Africa, Uruguay, BEUC, CI, ENCA, IBFAN, HKI, UNICEF, WFPHA.	*Causation: what or who is the cause of the problem and how is risk perceived?*
		• Adverse health outcomes, such as NCDs, could be the result of unhealthy packaged foods being excluded from requiring FOPNL. • When food products that are high in unhealthy nutrients do not have FOPNL, consumers will not see the simplified nutritional information that may help them make heathier choices. • Existing Codex standards do not adequately protect consumer health. • Codex fails to define some unhealthy packaged foods (eg, sports foods) which may exempt these products from requiring FOPNL. • Foods that are the sole source of nutrition should not require FOPNL as they have a special dietary purpose, although this could lead to unhealthy packaged foods for infants and young children being inappropriately promoted. • Incoherence between Codex standards, nutrient profiles, and international laws could inappropriately exclude foods for infants and young children from FOPNL.
		*Responsibility: who is responsible for addressing the problem?*
		• Codex standards and guidelines. • Nutrient profiles. • International laws (eg, WHO Code and WHA resolutions).
		*Solutions: what are the policy actions to mitigate the problem?*
		• All unhealthy packaged foods should require FOPNL, unless disputed by scientific evidence. • Codex standards and guidelines must provide better protection against unhealthy packaged foods via warning labels, particularly for infants and young children. • FOPNL could provide consumers with protection from foods that are not defined by Codex texts. • Foods for vulnerable groups should be added to the FOPNL Guidelines exclusion list. • FOPNL should not be excluded for foods for infants and young children simply because they are regulated by other policies.
		*What are the potential harms of those actions?*
		• FOPNL may be used to exploit some products by food corporations. • Codex regulations may not provide sufficient protection from nutrients of concern. • Incoherence of policies may affect harmonization of FOPNL schemes.
		*Evidence: what evidence is used to support this position?*
		• Codex standards and guidelines. • WHA resolutions, WHO Code, reports, and guidelines.^78-81^ • Global Burden of Disease Study.^82^
*Summary of public health interest themes of perceived risk or harm:* Exploitative marketing, strategic product positioning, systemic barriers, inadequate regulations, voluntary systems, commercial determinants of health, corporate scientific activities, conflicts of interest.		

Abbreviations: BEUC, Bureau Européen des Unions de Consommateurs; CCFL, Codex Committee on Food Labelling; CI, Consumers International; CXG 2-1985, Codex Guidelines on Nutrition Labelling; ENCA, European Network of Childbirth Associations; ESSNA: European Specialist Sports Nutrition Alliance; EU, European Union; FAO, Food and Agriculture Organization; FSDU, Foods for Special Dietary Uses; FOPNL, Front-of-pack Nutrition Labelling; HICs, high-income countries; HKI, Helen Keller International; HSR, Health Star Ratings; IACFO, International Association of Consumer Food Organizations; IBFAN, International Baby Foods Action Network; ICGMA, International Council of Grocery Manufacturers Associations; IDF/FIL, International Dairy Federation; IFU, International Fruit and Vegetable Juice Association; IFT, Institute of Food Technologists; ISDI, International Special Dietary Foods Industries; LICs, low-income countries; NCD, Non-communicable Disease; NZ, New Zealand; PAHO, Pan American Health Organization; UNICEF, United Nations Children’s Fund; US, United States; WFPHA, World Federation of Public Health Associations; WHA, World Health Assembly; WHO, World Health Organization; WHO Code, International Code of Marketing of Breast-milk Substitutes; WOF, World Obesity Foundation; WTO, World Trade Organization; eWG, Electronic Working Group.

###  Provision 1: Front-of-Pack Nutrition Labelling Schemes & Depiction of Nutrients

 The general aim of FOPNL “high in” nutritional descriptions was to simplify nutrition information or highlight the presence of unhealthy nutrients for consumers. This was highly contested by different actors and interests.

####  Commercial Interest Framing

 In response to Costa Rica’s discussion paper outlining production costs, trade impacts on businesses in developing countries and confusing multi-labelling schemes with major economic implications, an electronic working group was appointed to develop general FOPNL Guidelines.^51^

 The IDF/FIL and FoodDrinkEurope supported a request for the Guidelines to be “scientifically grounded,” “non-discriminatory,” and “facilitating [of] global food trade” to align with EU regulations (IDF/FIL, 2017 and FoodDrinkEurope, 2019). Dietary awareness was framed as the purpose of FOPNL with the focus on positive (healthy), as opposed to negative (unhealthy) ingredients:

 “*Labelling systems need to be complemented by education, awareness and communication to the consumer AND recommending a healthy diet based on positive aspects of packaged food eg, low in sodium, low in saturated fats, source of nutrients such as vitamins, minerals or essential fatty acids…to build a nutritional profile for labeling purposes, beyond a single definition of ‘high in’ fats, sugars and sodium” *(Costa Rica, Paraguay, 2017).

 The Institute of Food Technologists (IFT) framed warnings as “directive” and reinforced the view that the focus of FOPNL Guidelines should be “based on science” and “positive” aspects of packaged foods; “FOPNL should frame information as constructive nutritional help, not warnings. It should be meaningful and not alarmist or mis-leading to consumers” (IFT, 2021).

 Overall, presenting positive aspects of packaged foods was framed as the priority, instead of risks or harms to health. Consequently, the EU, 13 member states and six observers rejected isolated graphics and textual indications, such as “high-in saturated fat,” “high-in sugar,” and “high-in salt/sodium” warnings.^83^ Two observers shared some apprehensions:

 “*Graphics, verbiage or other depictions which could give rise to doubt about the safety of similar food or which could arouse or exploit fear in the consumer should not be adopted. They should also not lead to discrimination of other foods” *(International Fruit and Vegetable Juice Association [IFU], International Council of Grocery Manufacturers Associations, 2019).

 The International Special Dietary Foods Industries (ISDI) raised specific concerns for FSDU, warning that FOPNL “would unjustifiably discriminate against these categories and undermine the purpose of the products” (ISDI, 2021).

 Some industry groups promoted FOPNL that “take(s) into account the overall nutritional value and substantial scientific evidence for health benefits of a range of nutrient-rich foods with both beneficial and detrimental nutrients” (IDF/FIL, 2017). This was framed by the IFU and others as empowering consumer choice:

 “*It is necessary, that not only negative aspects can be shown on a FOPNL, but also positive (to make sure that consumers can make the informed food choice that reflects their dietary needs)” *(IFU, 2019).

 Some argued to ensure the FOPNL Guidelines remained broad in scope, for instance when the CCFL was reminded to “keep the principles high level and not too specific” as requested by the Chair (IDF/FIL, 2021).

####  Public Health Interest Framing

 Among criteria for a global standard for interpretive FOPNL, the IACFO considered how out-of-date Codex labelling standards failed to convey adequate nutritional information. However, despite having particular relevance to public health, FOPNL was assigned under an ambiguous agenda title—Other Business and Future Work,^77^ resulting in IACFO raising the following concern:

 “*Many government nutrition policy specialists are not attending the 2016 session of the CCFL because none of the agenda items pertain to nutrition and few relate even indirectly to human health” *(IACFO, 2016).

 An example of how public health actors framed risk or harm, was when IACFO highlighted the many FOPNL schemes available in different countries, for example, those prohibiting front-of-pack marketing claims and mandating warning labels. This was framed as particularly problematic when schemes used in LMICs were incoherent with those implemented in HICs:

 “*Recent reports of the WTO Technical Barriers to Trade Committee meetings reveal that there are differences of opinion between some of these countries and Canada, the United States, the European Union, and others about the concordance between, for example, nutrition ‘stop signs’ and warning labels with Codex Standards and WTO rules”* (IACFO, 2016).

 To address this, IACFO proposed overarching guidelines to “encourage and empower” member states to better serve public health interests and simultaneously resist efforts to challenge countries individually through WTO trade disputes (IACFO, 2016).

 Some participants framed concerns differently, noting that public health experts and the *WHO Report of the Commission on Ending Childhood Obesity*^78^ recommended interpretive labelling. Consumers International (CI) explained that:

 “*Warning labels such as ‘high in sugar’ are considered to be such ‘interpretive labels’… To ensure consistency and policy coherence, Codex documents should also consider such labels to be FOPNL” *(CI, 2019).

 Several member states agreed that this simplified approach had been used successfully in countries to enhance consumers’ understanding of unhealthy packaged foods.^84^ Others framed support for “high-in” schemes by intergovernmental organizations, as justification for what should be included in the Guidelines:

 “*FAO and PAHO have publicly lauded the ‘High In’ warning label put forth in Chile as an example of front-of-pack labelling that the rest of the world should follow. It is prudent for Codex to include such labels in the definition of FOPNL” *(CI, 2019).

 Along with applying FOPNL as an effective strategy to improve public health outcomes, some public health actors were troubled by design details that could encourage the use of misleading “health halos.”^85^ For example, IACFO framed concerns for FOPNL featuring positive nutrients as being outside the scope of the *WHO Guiding Principles and Framework Manual for Front-of-pack Labelling for Promoting Healthy Diets*^79^:

 “*The presence of information on positive nutrients (eg, fibre, vitamins and minerals) has been shown to greatly influence health perceptions of a product, suggesting that the display of information about positive nutrients should be excluded on FOPNL appearing on less healthful products” *(IACFO, 2021).

 Several civil society organizations framed simplified labelling that reduces consumer confusion and health risks, as a way to:

 “*Avoid the ‘halo effect’ on risky products by allowing room to determine the scope of FOPNL depending on the type of label adopted, for example by prohibiting positive endorsements on breast-milk substitutes or alcohol, and/or ensuring nutrient-specific warnings are applied to ‘sports’ beverages as well as foods for infants and young children” *(World Federation of Public Health Associations, 2019).

 These arguments reiterated earlier discussions about “confusion created by nutritionally selective marketing claims and weak, inconsistent nutrition criteria for such claims likely undermine public health” (IACFO, 2016). In addition, efforts to avoid health risks or harms associated with unhealthy packaged foods were framed as fruitless, unless FOPNL was a mandatory industry requirement. As CI stressed:

 “*The food industry is unlikely to comply with any voluntary FOPNL that highlights negative properties of products they manufacture and discourages their purchase by consumers” *(CI, 2021).

 Overall, public health actors acknowledged that Codex standards provided minimal nutritional information and therefore, simplified interpretive FOPNL Guidelines could educate consumers about unhealthy foods. Furthermore, overarching guidelines could prevent trade disputes. Yet there were concerns that nutrition discussions were not being prioritized at Codex and FOPNL could be used to inappropriately markets foods.

###  Provision 2: Front-of-Pack Nutrition Labelling – Packaged Foods to Be Excluded

 One of the most highly contested issues was deciding which standardized and non-standardized food products should require FOPNL.

####  Commercial Interest Framing

 Actors with commercial interests, viewed FOPNL for FSDU as potentially perilous since they are designed for consumers with special dietary needs who are often already under medical supervision. For example, two member states and industry actors argued that standardized food products, including processed cereal-based foods, canned baby foods, formula for weight control diets, and foods with strict compositional requirements, should be exempt from FOPNL.

 Additionally, ISDI framed excluding other types of FSDU (eg, gluten free foods) as deceptive and undermining a consumer’s ability to choose freely, and regulatory decisions made by nations. Australia’s argument for FOPNL to apply to foods for infants and young children was framed as a way of supporting consumer choice and assisting parents in making healthier selections; while mitigating risks associated with:

 “*…the growing market of manufactured ‘food for young children’ and nutrition content concerns of these foods. In Australia, some foods that are targeted towards young children have voluntarily applied the HSR [health star rating system] (eg, some breakfast cereals, [and] novelty shaped pasta)” *(Australia, 2021).

 The definition of sports foods generated some controversy with ISDI, the US, and NZ suggesting countries have different ideas about what constitutes “sports foods” and recommended national standards over international harmonization. This perspective was shared by the EU and industry group European Specialist Sports Nutrition Alliance (ESSNA) who suggested that these products were already covered by Codex labelling standards (EU, ESSNA, 2019).

 The overall commercial framing of foods to be excluded from FOPNL was in support of consumers exercising the freedom of choice to select products, including products not guided by Codex standards, based on personal dietary requirements, and for countries to define specialized products as they wish.

####  Public Health Interest Framing

 Generally, public health actors also framed arguments for FOPNL in terms of informing and educating consumers to avert adverse health outcomes associated with unhealthy packaged foods. They generally opposed exclusions except when science based and when Codex standards did not provide sufficient protection, particularly for infant and young child feeding.

 Canada, Kuwait and several civil society organizations emphasized that sports foods were widely consumed by the public, including children, and should not be excluded from FOPNL. This was reinforced by CI:

 “*They may contain significant quantities of nutrients that are associated with increased burdens of non-communicable diseases. They are products which are often consumed by the general population, not just athletes. Children can also consume these products regularly in spite of their often-high content of sugar” *(CI, 2019).

 Some civil society organizations argued that *“the relevant Codex standards…are not fully protective in addressing the most common nutrients of concern for diets*” (Helen Keller International [HKI], 2021). Canada agreed that some categories of FSDU, including solid foods for young children, gluten-free foods, and sports foods, should require FOPNL because:

 “*These foods can be high in sugars, sodium and/or fat and consumers should be aware in order to make an informed decision…sports foods or drinks are not defined in Codex texts and there may be a wide range of foods included in this category” *(Canada, 2019).

 Additional risks associated with unhealthy foods for infants and young children were raised by South Africa:

 “*Older infants and young children fed processed complementary foods risk dental caries, obesity and develop preferences for bland ‘white’ foods. Ultra- processed products invariably contain chemical additives to stabilize, emulsify, thicken, regulate acidity, and act as anti-oxidants etc” *(South Africa, 2021).

 However, applying FOPNL Guidelines to products for infants and young children was framed by the International Baby Foods Action Network (IBFAN) and the European Network of Childbirth Associations (ENCA) as a potential conduit for inappropriate promotion, and a threat to the *International Code of Marketing of Breast-milk Substitutes.*^80^ They argued that:

 “*Processed complementary food products and formulas for infants and young children should not have FOPNL… To effectively safeguard infant and young child health, it is preferable to have warnings on these products” *(IBFAN, ENCA, 2021).

 South Africa’s framing of introducing FOPNL for foods for infants and young children, was as a tool to apply warnings to unhealthy packaged foods:

 “*Public health nutrition policy promotes the consumption of healthy nutritious foods for optimal health and development as well as the development of lifelong preferences for healthy foods. FOPNL in these situations can act as a warning to consumers regarding the use of ultra-processed food products at a vulnerable stage of growth and development” *(South Africa, 2021).

 The United Nations Children’s Fund (UNICEF) and HKI supported these concerns, although UNICEF urged coherence between Codex standards and international guidelines on foods marketed for infants and young children, and warned it was “not a reason to categorically exclude such foods and products from all FOPNL systems, provided that policies are applied consistently” (UNICEF, 2021).Similarly, Canada framed exclusions from FOPNL as appropriate for foods when used as the sole source of nutrition for vulnerable groups under medical supervision (eg, infants and people requiring liquid diets).

 Overall, public health actors argued in support of using FOPNL as a tool to protect health, including the empowerment of LMIC governments to adopt warning labels and other schemes that were uncommon in HICs. The exception to this position was that FOPNL should not be used to promote unhealthy special dietary foods such as sports drinks and certain products for infants and young children. Moreover, Codex standards were framed as not providing sufficient protection against unhealthy nutrients.

####  Outcome

 At the close of CCFL46, the Committee did not confirm whether FOPNL would incorporate warnings, or be non-discriminatory, although “scientifically valid consumer research”^27^ was added as a FOPNL requirement. The Guidelines also specified that symbols, graphics, and text that provide information on the overall nutritional value of packaged foods and nutrients could be included. Strong support was received for FOPNL exclusions for the following standards— Infant Formula/Formula for Special Medical Purposes, Follow-up Formula, and *the Labelling of and Claims for Foods for Special Medical Purposes*—others were to be decided domestically. Subsequently, the Codex Committee on Nutrition and Foods for Special Dietary Uses was informed that the FOPNL Guidelines had been added as an Annex to the *Codex Guidelines on Nutrition Labelling*.^27^

## Discussion

 Our research aimed to understand the politics of food standard-setting in Codex, using the development of the new FOPNL Guidelines, as a case study. We achieved this by enumerating actors and actor types involved in relevant CCFL meetings, and by examining how these different actors and interests contested and framed key issues during the Guidelines development process.

 Similar to the findings of earlier studies,^20-23^ our quantitative analysis of member state and observer representation in CCFL meetings show a strong skew towards actors from HICs over those from middle-income and LICs, and much greater representation of industry actors relative to civil society and other actors in both member state delegations and as observers.^76^

 We noted a significant increase in the participation of all actors in 2021 when meetings were held virtually and the FOPNL Guidelines were nearing completion.

 We also found that the large majority of government delegates participating in the CCFL meetings were from non-health ministries (eg, agriculture, commerce, and trade) relative to health ministries, irrespective of country income category. Industry actors were strongly represented in the delegations of not just HICs, but also middle-income ones, which supports reports of food industry influence penetrating government decision-making and public health policy in key middle-income country food markets.^1,3,86^ Transnational food companies, for example Abbott, Coca-Cola, and Nestlé, were represented across multiple member state delegations and income categories, demonstrating the importance of FOPNL to their interests, and suggesting coordination across multiple country delegations simultaneously.

 While the mandate of Codex is to protect consumer health and facilitate international trade in food,^5^ there was a clear under-representation of actors with public health and consumer interests in standard-setting, with limited participation in government delegations irrespective of country-income category, and as observers. Civil society participation is not only important to generating better outcomes for public health in food standard-setting, but this also adds legitimacy to decisions, strengthens accountability, and ensures that important issues are raised in subsequent meetings, and publicly recorded for future analysis and consideration.^87,88^

 Our qualitative results show how actors representing corporate and trade interests framed specific revisions to the Guidelines in terms of enabling consumers to exercise “free choice” by providing “positive” nutritional information, and in contrast, framed “high-in” warnings as misleading, discriminatory, and alarmist.^89^ This framing is consistent with studies showing strong industry opposition to innovative and proven effective FOPNL schemes, such as the octagonal warning labels adopted throughout Latin America, which pose a significant threat to their commercial interests and their own preferred industry designed labelling schemes.^90,91^

 When addressing which packaged foods should require FOPNL, most actors with commercial interests framed product exclusions from FOPNL as dependent on compositional requirements, arguing that products (predominantly foods for special dietary uses) that were strictly regulated by Codex standards, needed no further guidance. Deviation from these food standards could potentially undermine previous recommendations and evidence referenced by industry actors during the development of standards and endorsed by Codex. However, this argument seemed dependent on whether a product would benefit commercially by FOPNL, or not. This framing aligned with “corporate scientific activities,”^92-94^ whereby research receives corporate sponsorship to generate favourable evidence, including for the use of industry preferred packaging claims and labelling schemes. Industry actors highlighted isolated nutrients to promote positive nutrients via FOPNL while ignoring dietary patterns,^95^ and exploited scientific-sounding terms to encourage selective focus on nutrients that conveyed to consumers a products’ healthfulness.^96^

 Keeping guidelines broad was a frame used to support the implementation of national regulations, a position which can enable rather than constrain product marketing.^97^ For instance, this would allow sports foods to be excluded from requiring FOPNL in some countries, regardless of a high unhealthy nutrient content. The only other option for industry—reformulation—presents added harms to population health and further processing of food products.^98^ These arguments illustrate how fundamental conflicts of interest within Codex decision-making processes shape the broader concepts that frame how member states and consumers perceive unhealthy packaged foods, such as ultra-processed foods, and how commercial actors and interests can adversely influence public health outcomes.^99^

 Our qualitative results also show how actors representing public health priorities, framed Codex standards and decision-making processes as inadequate in supporting public health. Consumer groups argued that overarching FOPNL Guidelines could provide consumers with simplified nutrition information, such as “high-in” sugar warnings, that could have a high public health impact and apply to various schemes across countries of different income categories, and thereby resist trade disputes.

 Risk or harm was framed by public health interest groups in terms of “positive” nutritional information as overshadowing the danger that unhealthy packaged foods pose to population health, the potential for LMICs to be penalized by HICs for implementing bold FOPNL schemes (eg, Latin America’s octagonal warning labels), and inappropriate FOPNL allocation for food products.

 Sometimes frames were used to scrutinize systemic failures and call for more balanced engagement with greater public health participation and impact how *risk* would be framed in future deliberations. Moreover, the exclusion of nutrition and human health agenda items ignored the dual mandate of Codex and removed the opportunity for public health groups to equally engage in deliberations and challenge industry rhetoric.^5^ This important detail was raised in a conference room document,^52^ yet averted scrutiny, as it was missing from the CCFL43 report.^59^ Nevertheless, consumer groups continued to frame arguments preferencing “high-in” warnings as interpretive labelling, and overarching guidelines to increase labelling coherence, as recommended by the WHO *Report of the Commission on Ending Childhood Obesity.*^78^

 Concerns were also raised by these groups over technical details used in some countries to exploit misleading “health halos” for unhealthy products.^85^ This highlighted the confusion created by selective marketing claims and the importance of clear and consistent regulatory criteria to limit the use of nutrient content or other claims to promote unhealthy packaged foods. However, there was apprehension by some civil society organizations that FOPNL schemes, other than warning labels, could be of benefit to some products, yet serve as an inappropriate promotional tool for other products. Foods marketed to infants and young children, for example infant and follow-up formulas, would be in violation of WHO Guidance.^100^ A common theme shared by these actors, was that Codex standards fell short of protecting consumers from common nutrients of concern, such as the permissible sugar content in the *Standard for Processed Cereal-based Foods for Infants and Young Children*.^101^ Another concern was that FOPNL could be used to cross-promote products for infants and young children, which is prohibited by the *International Code of Marketing of Breast-milk Substitutes*.^80,102^ However, it was agreed that in most cases, consumers would benefit from the useful and interpretative guidance that FOPNL could provide.

 Overall, this study supports research that shows how harms to public health are downplayed and commercial and trade considerations are prioritized in Codex committee deliberations.^20-23,32,103^ This study also reiterates that Codex largely interprets *risk* as meaning lowering the probability of microbiological, chemical, and toxicological contamination, by recommending permissible levels of additives, pesticide residues, and nutrients.^5^ To-date, Codex has not equally prioritized the measures of *harm* raised by advocates for public health during CCFL proceedings, ie, protection from developing chronic diseases, such as cancer, diabetes, cardiovascular disease, obesity, and others. Hopefully, this will be addressed in the *Codex Strategic Plan 2020-2025 *under Codex’s contribution to Sustainable Development Goal 3, “ensuring healthy lives and promoting well-being for all, at all ages”^104^ (p. 5).

 This study also suggests that any attempt to reform the international food regulatory system, centred on Codex structures, standards and processes, is likely to be highly politicized. The lengthy deliberations over intricate technical aspects of the General FOPNL Guidelines, and regular acceptance of proposals by Codex that have been justified and dominated by corporate interests, compromise progression towards healthier and more sustainable diets. Nonetheless, this guidance is important because it informs policy when countries decide whether to embrace or reject regulatory options that facilitate the proliferation of unhealthy packaged foods.

###  Strengths and Limitations

 The findings from this paper have provided insights into the complex politics of food standard-setting, including conflicting interests between commerce and public health, and outcomes determined by the more powerful actors and prevailing commercial interests.

 Some limitations were encountered. To confirm validity, our framework could benefit from testing in other national or international food regulatory fora to analyze debates between commercial and public health interests. In addition, as conference room documents were not fully considered at CCFL meetings, nor required to be included in final reports,^61^ the correlation between our findings and final draft guidance outcomes may appear unclear. Developments were also often described in general terms without country or observer identification in committee discussion papers and reports. Greater context, specification, and validation of prevailing actor interests could have otherwise been provided by the authors. For example, the electronic working group and CCFL reported that 12 countries supported “warnings” as part of FOPNL and 14 countries (including the EU) did not.^57,105^ In this instance, disclosure was important because FOPNL schemes were being discussed that would determine whether consumers would be warned about unhealthy aspects of foods, or not. Furthermore, time constraints prevented some FOPNL aspects relevant to unhealthy packaged foods and environmental considerations from being examined, such as sustainability labelling claims, and the principles and criteria for food labelling exemptions in emergencies, which could relax or temporarily exempt labelling requirements.

## Conclusion

 Food systems in many countries are increasingly dominated by unhealthy packaged foods posing a major challenge for public health. For over 60 years, Codex has been influenced by specific actors which has resulted in policies that favour commerce and trade priorities over other considerations. This has enabled transnational food corporations to flourish, especially those that profit from selling unhealthy packaged foods. However, as this form of governance has traditionally embraced a narrow perception of *risk*—food safety risk to an individual—considerably less emphasis has been placed on wider risks to population health.

 Our study shows that Codex FOPNL Guidelines are disproportionately influenced by arguments framed by HICs, non-health ministries, and corporations aligned with the promotion of international sales and trade. There is comparatively much less engagement by lower-income countries, health ministries, and civil society organizations. Therefore, public health interests are not represented anywhere near equally as commercial interests in Codex deliberations. This likely limits the ability of governments to implement food regulations that will generate positive long-term public health and environmental outcomes.

 As such, we suggest that Codex members strengthen ministry of health and civil society representation within their delegations and for Codex to lead the way by countering the asymmetry in participation based on country income level and actor type, and the conflicts of interest that enable commercial interests to powerfully inform standard-setting. In doing so, member states and Codex can play proactive roles in the sustainable food systems transformation agenda.

## Ethical issues

 Ethics approval was not required as the study used secondary data only.

## Conflicts of interest

 Authors declare that they have no conflicts of interest.

## Disclaimer

 ML is a member of the Food Standards Australia New Zealand (FSANZ) Board, the views expressed in this paper do not necessarily represent the views, decisions, or policies of the FSANZ Board.

## Supplementary files

Supplementary file 1. Document Search - Inclusion and Exclusion Criteria.

Supplementary file 2. Main Industry Actors Representing Member States During Deliberations Under the Codex Committee on Food Labelling (2016-2023).

Supplementary file 3. Industry and Civil Society Actors as NGO Observers Listed as Participants Under the Codex Committee on Food Labelling (2016-2023).
