# Supplementary material for: Understanding the Politics of Food Regulation and Public Health: An Analysis of Codex Standard-Setting Processes on Food Labelling
Source: Int J Health Policy Manag. 2024 Oct 14;13:8310. doi: 10.34172/ijhpm.8310 (PMC11549567; doi:10.34172/ijhpm.8310)
Supplement: Supplementary file 1 — Document Search - Inclusion and Exclusion Criteria. [file ijhpm-13-8310-s001.pdf]

Article title: Understanding the Politics of Food Regulation and Public Health: An Analysis of Codex Standard-Setting Processes on Food Labelling

Journal name: International Journal of Health Policy and Management (IJHPM)

Authors' information: Monique Boatwright[1]*, Mark Lawrence[2], Angela Carriedo[3], Scott Slater[4], David McCoy[5], Tanita Northcott[4], Phillip Baker[1]

[1]Sydney School of Public Health, Faculty of Medicine and Health, University of Sydney, Sydney, NSW, Australia.

[2]Institute for Physical Activity and Nutrition, School of Exercise and Nutrition Science, Deakin University, Geelong, VIC, Australia.

[3]Department of Health, University of Bath, Bath, UK.

[4]School of Exercise and Nutrition Science, Deakin University, Geelong, VIC, Australia.

[5]International Institute for Global Health, United Nations University, Kuala Lumpur, Malaysia.

***Correspondence to:** Monique Boatwright; Email: monique.boatwright@sydney.edu.au

Citation: Boatwright M, Lawrence M, Carriedo A, et al. Understanding the politics of food regulation and public health: an analysis of Codex standard-setting processes on food labelling. Int J Health Policy Manag. 2024;13:8310.doi:10.34172/ijhpm.8310

Supplementary file 1. Document Search - Inclusion and Exclusion Criteria

Table S1. Inclusion and exclusion criteria applied in the document search process.

Inclusion Criteria	Exclusion Criteria
CCFL documents (agendas, session reports, comments, CRDs, electronic working group reports, and circular letters) and 2015 CCFL documents filed under CCFL43 (n=247)	Documents outside of 2016-2023 (other than those under CCFL43)
CCFL agendas, background documents, proposals, discussion papers and reviews relating to the new Guidelines for FOPNL (n=24)	Documents under the purview of committees other than the CCFL
CCFL circular letters and reports referring to FOPNL (n=9)	CCFL information documents and EWG invitation forum documents
CCFL comments by member state delegates and observers referring to FOPNL (n=32)	Documents not in English (n=5)
CCFL comments by member state delegates and observers focused on labelling principles for 'high-in' warnings on FOPNL and FOPNL inclusion/exclusion criteria for unhealthy packaged foods (n=19)	CCFL documents referencing FOPNL for alcoholic beverages, and principles and criteria for food labelling regarding exemptions in emergencies, sustainability claims, allergens, internet sales, e-commerce, and non-retail containers (n=182)
Documents included (n=60)	

Abbreviations: CCFL, Codex Committee on Food Labelling; CRDs, Conference Room Documents; EWG, Electronic Working Group; FOPNL, Front-of-pack Nutrition Labelling.
