# Supplementary material for: Understanding the Politics of Food Regulation and Public Health: An Analysis of Codex Standard-Setting Processes on Food Labelling
Source: Int J Health Policy Manag. 2024 Oct 14;13:8310. doi: 10.34172/ijhpm.8310 (PMC11549567; doi:10.34172/ijhpm.8310)
Supplement: Supplementary file 2 — Main Industry Actors Representing Member States During Deliberations Under the Codex Committee on Food Labelling (2016-2023). [file ijhpm-13-8310-s002.pdf]

Article title: Understanding the Politics of Food Regulation and Public Health: An Analysis of Codex Standard-Setting Processes on Food Labelling

Journal name: International Journal of Health Policy and Management (IJHPM)

Authors' information: Monique Boatwright[1]*, Mark Lawrence[2], Angela Carriedo[3], Scott Slater[4], David McCoy[5], Tanita Northcott[4], Phillip Baker[1]

[1]Sydney School of Public Health, Faculty of Medicine and Health, University of Sydney, Sydney, NSW, Australia.

[2]Institute for Physical Activity and Nutrition, School of Exercise and Nutrition Science, Deakin University, Geelong, VIC, Australia.

[3]Department of Health, University of Bath, Bath, UK.

[4]School of Exercise and Nutrition Science, Deakin University, Geelong, VIC, Australia.

[5]International Institute for Global Health, United Nations University, Kuala Lumpur, Malaysia.

***Correspondence to:** Monique Boatwright; Email: monique.boatwright@sydney.edu.au

Citation: Boatwright M, Lawrence M, Carriedo A, et al. Understanding the politics of food regulation and public health: an analysis of Codex standard-setting processes on food labelling. Int J Health Policy Manag. 2024;13:8310.doi:10.34172/ijhpm.8310

Supplementary file 2. Main Industry Actors Representing Member States During Deliberations Under the Codex Committee on Food Labelling (2016-2023)

Table S2. Main industry actors in member state delegations during Codex Committee on Food Labelling meetings from 2016 to 2023

	Year industry represented country delegations	Abbott	Alimentos y Bebidas de Chile (AB Chile)	Associação Brasileira da	Acepalma	Ajegroup	Ajinomoto	Amazon	ANPRAC	Association of the German Dairy Industry (MIV)	Barkath CORO Manufacturing	Bavarian Dairy Industrie Co-op	Bel Group	Reckitt Benckiser	Brenntag Vietnam Ltd.	Cámara Costarricense de la Industria Alimentaria (CACIA)	Camara Panamena de Alimentos	Cámara Nacional de la Industria de Conservas Alimenticias (CANAINCA)	Cenipalma	Cenosud Retail	Centro de la Industria Lechero	China National Food Industry Association	Centro de la Industria Lechera (CIL)	The National Industry Confederation (CNI)
**High-income country delegations**																								
Australia	2019, 2021, 2023																							
Canada	2016, 2017																							
Chile	2017, 2019, 2021		3																	1				
France	2016																							
Germany	2016, 2017, 2019									2		1												
Hungary	2021																							
Italy	2016, 2019, 2021	5																						
Japan	2016, 2023																							
New Zealand	2019, 2021, 2023																							
Panama	2021																1							
Republic of Korea	2017																							
Switzerland	2016, 2017, 2019																							
United States	2016, 2017, 2021, 2023							1						3										
Uruguay	2021																							
**Middle-income country delegations**																								
Argentina	2017, 2019, 2021																				1		1	
Brazil	2017, 2019, 2021, 2023	2		2			1							2										1
China	2021																					1		
Colombia	2017, 2019	6			1														1					
Costa Rica	2016, 2017, 2019															5								
Egypt	2016, 2023												2											
Guyana	2021																							
India	2016, 2017, 2019, 2021, 2023																							
Iran	2021, 2023																							
Kenya	2021																							
Malaysia	2016, 2021, 2023										1													
Mexico	2016, 2019, 2021	4							1									1						
Morocco	2021, 2023																							
Nigeria	2016, 2017, 2019, 2023																							
Peru	2021					1																		
Philippines	2021																							
Russian Federation	2016,																							
Thailand	2016, 2019, 2021, 2023																							
Vietnam	2021														2									
**Low-income country delegations**																								
Madagascar	2023																							
Mozambique	2016																							
Rwanda	2021																							
Syrian Arab Republic	2021																							

	Year industry represented country delegations	Coca-Cola	Conamin	ConMéxico (Coca-Cola, Unilever, Nestlé)	CONCAMIN	Corn Refiners	Danone	Effecta Foods & Care SAC	Federalimentare	Federation of Malaysian Manufacturers (DSM)	Ferrero	FICOPAM	FMM MAFMAG	Fonterra	Food and Grocery Council	Friesland Campina	Guyana Marketing Corporation	International Life Sciences Institute	Japan Soft Drink Assoc.	Korea Culinary Science Institute	Korea Food Research Institute	Laboratoire Officiel d'Analyses et de Reserches Chiminques	Laboratoria Tecnologico del Uruguay
**High-income country delegations**																							
Australia	2019, 2021, 2023	3																					
Canada	2016, 2017																						
Chile	2017, 2019, 2021	2																					
France	2016						1	1															
Germany	2016, 2017, 2019	1																					
Hungary	2021																						
Italy	2016, 2019, 2021								1	1	2												
Japan	2016, 2023																	1	1				
New Zealand	2019, 2021, 2023													4	1								
Panama	2021																						
Republic of Korea	2017																			1	4		
Switzerland	2016, 2017, 2019																						
United States	2016, 2017, 2021, 2023					2																	
Uruguay	2021																						1
**Middle-income country delegations**																							
Argentina	2017, 2019, 2021	1																					
Brazil	2017, 2019, 2021, 2023																						
China	2021																						
Colombia	2017, 2019																						
Costa Rica	2016, 2017, 2019																						
Egypt	2016, 2023	1																					
Guyana	2021																1						
India	2016, 2017, 2019, 2021, 2023	1																					
Iran	2021, 2023																						
Kenya	2021																						
Malaysia	2016, 2021, 2023									1			1										
Mexico	2016, 2019, 2021	2	1	1	1																		
Morocco	2021, 2023	1										1										1	
Nigeria	2016, 2017, 2019, 2023	4																					
Peru	2021							1															
Philippines	2021																						
Russian Federation	2016,																						
Thailand	2016, 2019, 2021, 2023																						
Vietnam	2021															1							
**Low-income country delegations**																							
Madagascar	2023																						
Mozambique	2016																						
Rwanda	2021																						
Syrian Arab Republic	2021																						

	Year industry represented country delegations	Malaysia Palm Oil Council	Mars	MINIMEX	National Dairy Development Board	National Institute of Pharmacy and Nutrition	National Seasoning Manufacturers Assoc.	Nestlé	Nutrition & Biosciences	Pacific Organic Seafood Assoc.	Palm Oil Board	Philippine Association of Food Technologists	Private Sector	Russian Union of Industrialists and Entrepreneurs (RUIE)	Syndicat des Industries de Madagascar (SIM)	Servicios Integrales Panamá (SIPA)	Sociedad Nacional de Industrias	Sindyan Company for Food Manufacturing	TES Nutrihealth Strategic Consultancy	Thai Frozen Foods Association	The Federation of Thai Industries	Thai Food Processors Assoc.	Unilever
**High-income country delegations**																							
Australia	2019, 2021, 2023																						
Canada	2016, 2017									1													1
Chile	2017, 2019, 2021							1															
France	2016																						
Germany	2016, 2017, 2019																						
Hungary	2021					3																	
Italy	2016, 2019, 2021																						
Japan	2016, 2023																						
New Zealand	2019, 2021, 2023																						
Panama	2021															1							
Republic of Korea	2017																						
Switzerland	2016, 2017, 2019							3															
United States	2016, 2017, 2021, 2023		1				1	1															
Uruguay	2021																						
**Middle-income country delegations**																							
Argentina	2017, 2019, 2021																						
Brazil	2017, 2019, 2021, 2023																						
China	2021																						
Colombia	2017, 2019																						
Costa Rica	2016, 2017, 2019																						
Egypt	2016, 2023																						
Guyana	2021																						
India	2016, 2017, 2019, 2021, 2023				5																		
Iran	2021, 2023												3										
Kenya	2021							1															
Malaysia	2016, 2021, 2023	2									4								1				
Mexico	2016, 2019, 2021							1															
Morocco	2021, 2023							1															
Nigeria	2016, 2017, 2019, 2023																						
Peru	2021							1									1						
Philippines	2021											1											
Russian Federation	2016,													2									
Thailand	2016, 2019, 2021, 2023																			5	5	5	
Vietnam	2021								2														
**Low-income country delegations**																							
Madagascar	2023														1								
Mozambique	2016							1															
Rwanda	2021			1																			
Syrian Arab Republic	2021																	1					

Note: The numbers within each highlighted cell represents the number of times an industry actor participated in a member state delegation
