# Supplementary material for: Understanding the Politics of Food Regulation and Public Health: An Analysis of Codex Standard-Setting Processes on Food Labelling
Source: Int J Health Policy Manag. 2024 Oct 14;13:8310. doi: 10.34172/ijhpm.8310 (PMC11549567; doi:10.34172/ijhpm.8310)
Supplement: Supplementary file 3 — Industry and Civil Society Actors as NGO Observers Listed as Participants Under the Codex Committee on Food Labelling (2016-2023). [file ijhpm-13-8310-s003.pdf]

Article title: Understanding the Politics of Food Regulation and Public Health: An Analysis of Codex Standard-Setting Processes on Food Labelling

Journal name: International Journal of Health Policy and Management (IJHPM)

Authors' information: Monique Boatwright[1]*, Mark Lawrence[2], Angela Carriedo[3], Scott Slater[4], David McCoy[5], Tanita Northcott[4], Phillip Baker[1]

[1]Sydney School of Public Health, Faculty of Medicine and Health, University of Sydney, Sydney, NSW, Australia.

[2]Institute for Physical Activity and Nutrition, School of Exercise and Nutrition Science, Deakin University, Geelong, VIC, Australia.

[3]Department of Health, University of Bath, Bath, UK.

[4]School of Exercise and Nutrition Science, Deakin University, Geelong, VIC, Australia.

[5]International Institute for Global Health, United Nations University, Kuala Lumpur, Malaysia.

***Correspondence to:** Monique Boatwright; Email: monique.boatwright@sydney.edu.au

Citation: Boatwright M, Lawrence M, Carriedo A, et al. Understanding the politics of food regulation and public health: an analysis of Codex standard-setting processes on food labelling. Int J Health Policy Manag. 2024;13:8310.doi:10.34172/ijhpm.8310

Supplementary file 3. Industry and Civil Society Actors as NGO Observers Listed as Participants Under the Codex Committee on Food Labelling (2016-2023).

Table S3. Industry and civil society actors as NGO observers listed as participants under the Codex Committee on Food Labelling (2016-2023).

**Industry actors as NGO observers**	**Year of CCFL report**
Alianza Latinoamericana de Asociaciones de la Industria de Alimentos Y Bebidas (ALAIAB)	2023
Association of European Coeliac Societies (AOECS)	2021, 2023
Calorie Control Council (CCC)	2021, 2023
Comite Europeen Des Fabricants De Sucre (CEFS)	2021, 2023
Council for Responsible Nutrition (CRN)	2021, 2023
European Cocoa Association	2021
European Federation of Allergy and Airways Diseases Patients' Associations (EFA)	2021, 2023
Federation Internationale Des Vins Et Spiritueux (FIVS)	2017, 2019, 2021, 2023
Food Industry Asia (FIA)	2017, 2019, 2021, 2023
FoodDrinkEurope	2016, 2017, 2019, 2021, 2023
Global Organization for EPA and DHA Omega-3S (GOED)	2019
Good Food Institute (GFI)	2021
Institute of Food Technologists (IFT)	2016, 2017, 2019, 2021, 2023
International Alliance of Dietary/Food Supplement Associations (IADSA)	2016, 2021, 2023

International Chewing Gum Association (ICGA)	2016, 2017, 2019, 2021, 2023
International Confectionary Association (ICA/IOCCC)	2019, 2021
International Co-operative Alliance (ICA)	2016, 2017, 2019, 2021, 2023
International Council of Beverages Associations (ICBA)	2016, 2017, 2019, 2021, 2023
International Council of Grocery Manufacturers Associations (ICGMA)	2016, 2017, 2019, 2021, 2023
International Council on Amino Acid Science (ICAAS)	2016, 2017, 2019, 2021
International Dairy Federation (IDF/FIL)	2016, 2017, 2019, 2021, 2023
International Federation of Organic Agriculture Movements (IFOAM)	2016
International Food Additives Council (IFAC)	2016
International Fruit and Vegetable Juice Association (IFU)	2019, 2021, 2023
International Glutamate Technical Committee (IGTC)	2019, 2021
International Life Sciences (ILSI)	2016, 2021
International Meat Secretariat (IMS)	2021
International Nut and Dried Fruit Council Foundation (INC)	2016
International Organization of the Flavour Industry (IOFI)	2016, 2017
International Special Dietary Foods Industries (ISDI)	2017, 2019, 2021, 2023
International Union of Food Science and Technology (IUFOST)	2023
Safe Supply of Affordable Food Everywhere (SSAFE)	2017, 2019, 2021
The European Margarine Association (IMACE)	2021
World Processing Tomato Council (WPTC)	2021
**CSO actors as NGO observers**	**Year of CCFL report**
International Baby Food Action Network (IBFAN)	2016, 2019, 2021
International Association of Consumer Food Organization (IACFO)	2016, 2017, 2021, 2023
Consumers International (CI)	2019, 2021
European Network of Childbirth Associations (ENCA)	2019,
Helen Keller International (HKI)	2019, 2021
National Health Federation (NHF)	2019, 2021
World Federation of Public Health Associations (WFPHA)	2019, 2021
World Obesity Federation (WOF)	2019, 2021, 2023
European Federation of the Associations of Dietitians (EFAD)	2021
European Alcohol Policy Alliance (EUROCARE)	2021
International Lactation Consultant Association (ILCA)	2021
European Network of Childbirth Associations (ENCA)	2023

Abbreviations: CCFL, Codex Committee on Food Labelling; CSO, Civil Society Organization; NGO, Non-Governmental Organization.
